# Supplementary material for: Mutations in PCYT2 disrupt etherlipid biosynthesis and cause a complex hereditary spastic paraplegia
Source: Brain. 2019 Oct 22;142(11):3382–97. doi: 10.1093/brain/awz291 (PMC6821184; doi:10.1093/brain/awz291)
Supplement: awz291_Supplementary_Data [file awz291_supplementary_data.zip › awz291-Suppl_data/Supplementary_Data4.zip!/Supplementary information 4 - plasma lipidomics.zip!/Plasma lipidomics/Control vs Patient2/Statistical plots and data/Heatmap sorted on FDR.pdf]

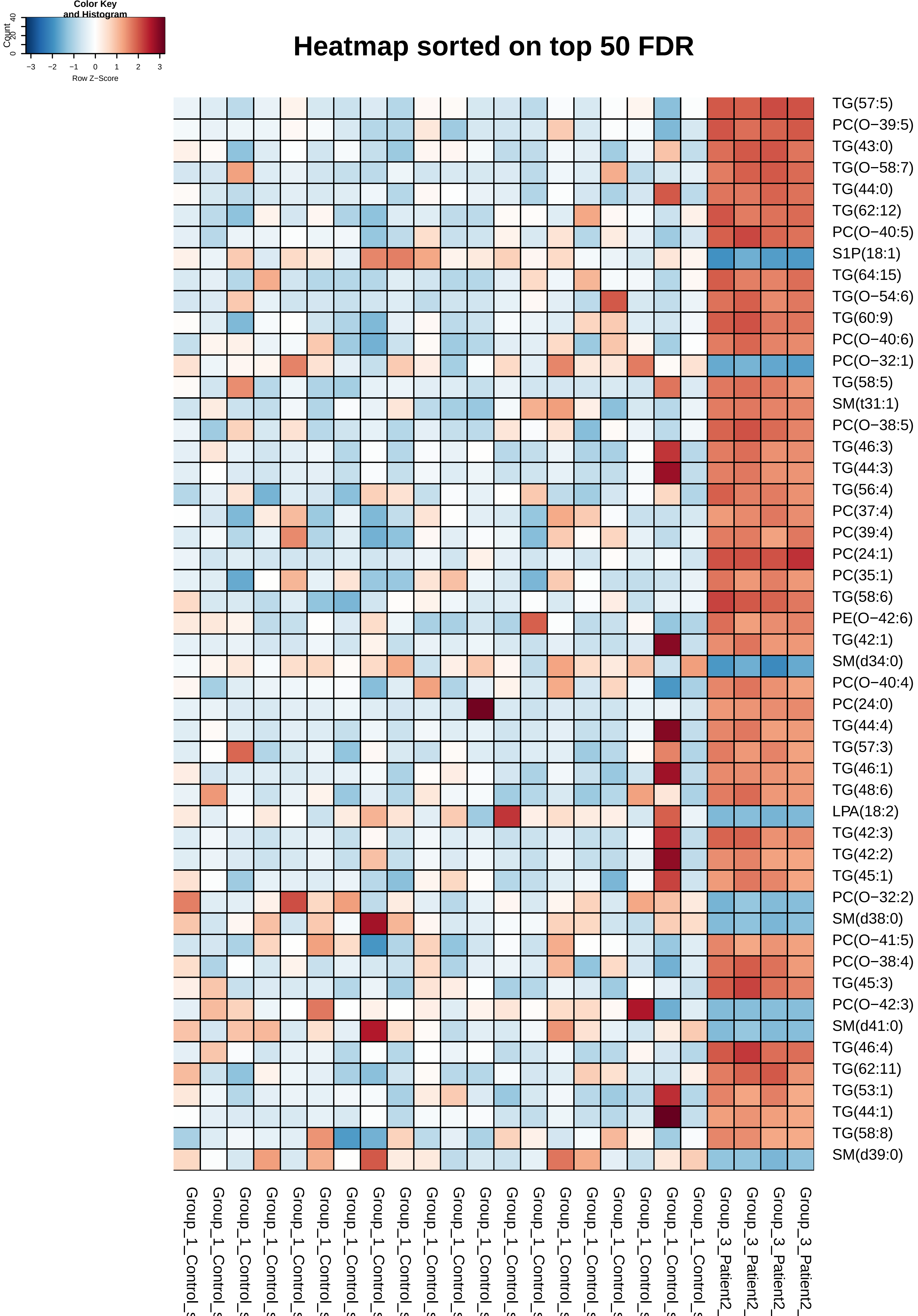

Color Key and Histogram
Count
Row Z−Score
Heatmap sorted on top 50 FDR
TG(57:5)
PC(O−39:5)
TG(43:0)
TG(O−58:7)
TG(44:0)
TG(62:12)
PC(O−40:5)
S1P(18:1)
TG(64:15)
TG(O−54:6)
TG(60:9)
PC(O−40:6)
PC(O−32:1)
TG(58:5)
SM(t31:1)
PC(O−38:5)
TG(46:3)
TG(44:3)
TG(56:4)
PC(37:4)
PC(39:4)
PC(24:1)
PC(35:1)
TG(58:6)
PE(O−42:6)
TG(42:1)
SM(d34:0)
PC(O−40:4)
PC(24:0)
TG(44:4)
TG(57:3)
TG(46:1)
TG(48:6)
LPA(18:2)
TG(42:3)
TG(42:2)
TG(45:1)
PC(O−32:2)
SM(d38:0)
PC(O−41:5)
PC(O−38:4)
TG(45:3)
PC(O−42:3)
SM(d41:0)
TG(46:4)
TG(62:11)
TG(53:1)
TG(44:1)
TG(58:8)
SM(d39:0)
Group_1_Control_s
Group_3_Patient2_
